# Assessment of Serum Free Light Chains as a Marker of Diabetic Nephropathy; A Cross-Sectional Study in the Kumasi Metropolis

**DOI:** 10.3389/fcdhc.2022.881202

**Published:** 2022-08-09

**Authors:** Elizabeth Sorvor, William K. B. A. Owiredu, Perditer Okyere, Max Efui Annani-Akollor, Sampson Donkor, Richard Bannor, Felix B.K. Sorvor, Richard K.D. Ephraim

**Affiliations:** ^1^ Department of Molecular Medicine, Kwame Nkrumah University of Science and Technology, Kumasi, Ghana; ^2^ Suntreso Government Hospital, Kumasi, Ghana; ^3^ Department of Internal Medicine, Kwame Nkrumah University of Science and Technology, Kumasi, Ghana; ^4^ Komfo Anokye Teaching Hospital, Kumasi, Ghana; ^5^ Department of Allied Health Sciences, University of Connecticut, Storrs, United States; ^6^ UConn Center for mHealth and Social Media, Institute for Collaboration on Health, Intervention, and Policy, University of Connecticut, Storrs, United States; ^7^ National Tuberculosis Control Programme, Accra, Ghana; ^8^ Department of Medical Laboratory Science, University of Cape Coast, Cape Coast, Ghana

**Keywords:** free light chains, diabetic nephropathy, predictive value, diagnostic tool, diabetes mellitus

## Abstract

**Aims:**

Although traditional tests such as serum urea, creatinine, and microalbuminuria have been widely employed in the diagnosis of diabetic nephropathy, their sensitivity and accuracy are limited because kidney damage precedes the excretion of these biomarkers. This study investigated the role of serum free light chains in the disease manifestation of diabetic nephropathy.

**Materials and Methods:**

Using a cross-sectional design we recruited 107 diabetes mellitus out-patients who visited the Diabetes and Renal Disease Clinics at the Komfo Anokye Teaching Hospital, Manhyia District Hospital, and Suntreso Government Hospital all in Ghana from November 2019 to February 2020. Five (5) mls of blood was collected from each participant and analyzed for fasting blood glucose (FBG) urea, creatinine, immunoglobulin free light chains. Urine samples were obtained and analyzed for albumin. Anthropometric characteristics were also measured. Data were analyzed using descriptive analysis, analysis of variance (ANOVA) test, Tukey HSD *post hoc*, and Kruskal Wallis test. Chi-squared test was used to examine if there are significant associations with the indicators of interest. In addition, Spearman’s correlation was used to test for associations between appropriate variables. Receiver operating characteristic analysis (ROC) was also performed to assess the diagnostic performance of free light chains.

**Results:**

The mean age of studied participants was 58.2 years (SD: ± 11.1), 63.2% were females and most of the participants were married (63.0%). The mean FBG of the studied participants was 8.0mmol/L (SD: ± 5.86), and the average duration of diabetes mellitus (DM) was 11.88 years (SD: ± 7.96). The median serum Kappa, Lambda, and Kappa: Lambda ratios for the studied participants were 18.51 (15.63-24.18), 12.19(10.84-14.48), and 1.50(1.23-1.86) respectively. A positive correlation was observed between albuminuria and; Kappa (rs=0.132; p=0.209), and Lambda (rs=0.076; p=0.469). However, a negative correlation was observed between albuminuria and K: L ratio (rs=-0.006; p=0.956).

**Conclusions:**

The current study observed an increasing trend in the levels of free light chains and degree of diabetic nephropathy, although not statistically significant. The exploration of serum free light chains as a better marker of diabetic nephropathy showed very promising results but further studies are required to elucidate its predictive value as a diagnostic tool for diabetic nephropathy.

## Introduction

Diabetic nephropathy is one of the most significant complications of diabetes mellitus and it is known to be responsible for about 40-50% of the cases of end-stage kidney failure (1). The prevalence of diabetic nephropathy is increasing with the diabetes mellitus epidemic; one-third to half of the patients with diabetes mellitus develop kidney problems (2). Diabetic nephropathy is linked with end-stage kidney failure, cardiovascular disease, and increased healthcare costs (3). Diabetic nephropathy is more prevalent among patients with diabetes mellitus in Africa and Ghana for that matter (4, 5) compared with their counterparts in the developed world (2) as a result of delayed diagnosis, limited screening, and diagnostic resources, poor glycaemic control and inadequate treatment at an early stage (6, 7).

Although diabetic nephropathy has been traditionally known to be a non-immune disease, recent studies have shown that immunity and inflammation are vital factors in its development and progression (8, 9). Free light chains are low molecular weight proteins and by-products of immunoglobulin synthesis (10–12). Free light chains are produced in excess during immunoglobulin synthesis and are cleared from the serum by the kidneys; therefore a decrease in glomerular filtration rate leads to an increase in free light chains (12). There are two forms of free light chains namely, kappa(k) and lambda(λ) (13). The serum concentration of free light chains depends on the balance between production and clearance by the kidneys (13). Free light chains have been shown to have a stronger relationship with renal inflammation than other inflammation markers like CRP (13–15).

Although microalbumin has been widely employed in the diagnosis of diabetic nephropathy, its sensitivity and accuracy are limited because kidney damage precedes albumin excretion (8, 16). Recent studies have demonstrated that the free light chain is a better marker for the risk of developing kidney damage than other markers of kidney function (10). A study conducted in the United Kingdom demonstrated that serum concentrations of free light chains increased progressively with chronic kidney disease (CKD) stage and also correlated strongly with renal function tests including cystatin C (17). Another study carried out in the UK among type two diabetes mellitus patients indicate significantly elevated levels of free light chains (FLC) before the development of overt renal impairment, signifying their possible role in predicting early diabetic nephropathy (13, 18, 19).

Although some studies have been conducted to explore the efficacy of free light chains in the diagnosis of kidney diseases, only a few have been done in Africa and for that matter Ghana. There is also limited information on this marker with diabetic nephropathy. This current study, therefore, seeks to investigate serum free light chain as a marker in the diagnosis of diabetic nephropathy among diabetes mellitus patients in the Ghanaian context.

## Materials and Methods

### Study Design, Sampling Technique, Study Area, and Population

This cross-sectional study was conducted from November 2019 to February 2020. The study comprised a total of 107 diabetes mellitus patients aged between eighteen (18) to eighty-five years (85). The study was conducted in three major hospitals in the Kumasi metropolis; Komfo Anokye Teaching Hospital (KATH), Suntreso Government Hospital, and Manhyia Government Hospital. The sample population included diabetes mellitus out-patients without nephropathy, and clinically diagnosed diabetic nephropathy patients attending the Diabetes Clinic and Renal Disease Clinics in the hospitals listed as well as those who qualified per the inclusion criteria and who consented to be part of the study. All diabetic and non-diabetic patients who did not meet the age criteria and other characteristics of the study and did not consent to the study were excluded from the study. In addition, all study participants who had recent infections, as well as multiple myeloma, were excluded from the study.

Sample size calculation

The following formula was used to estimate the sample size for the study.


$$n=\frac{Z^{2}p(1-p)}{d^{2}}$$

Where:

n = sample size

Z = 1.96 is the standard score for the confidence interval of 95%; p = expected prevalence; d = allowable margin of error = 5% or 0.05. According to a recent unpublished study carried out in the Ashanti region of Ghana, the prevalence of diabetic nephropathy is 11%. Substituting these values into the equation gives a sample size of 150 study participants. To provide for a 10% nonresponse rate, 165 participants were recruited for the study. Of the 165 eligible participants recruited using non randomized purposive sampling, 107 finally participated and provided complete data. Fifty eight (58) patients could not participate due to their inability to provide complete set of data (36) and not giving informed consent (22).

### Data Collection

A validated questionnaire was administered to obtain socio-demographic data including age, marital status, sex, education, and occupation from each participant. Information on the medical history including duration and family history of diabetes mellitus as well as side effects of the regular medication of the participants was also collected using their medical records. Anthropometric variables such as height, weight, waist, and hip circumference were measured using a tape measure and an electronic weighing scale, and BMI was calculated. Blood pressure was measured using a sphygmomanometer.

### Blood and Urine Collection and Laboratory Assessments

5ml of blood was collected from each participant through a venipuncture into gel separator tubes. The gel separator tube was centrifuged to obtain the serum and then further aliquoted into 1 ml Eppendorf tubes and stored at -20°C until assayed. In addition, spot urine samples were collected from study participants. The urine samples were analyzed for albumin.

Serum kappa(ĸ) and Lambda(ʎ) free light chain concentrations were measured using the ELISA technique manufactured by Melson Shanghai chemical Ltd, China. The reference interval used was ĸ 3.3 -19.4mg/L; ʎ 5.7 -26.3mg/L and ĸ/ʎ 0.26 -165 (19). Creatinine and urea were analyzed using the BT 1500 automated chemistry analyzer (Biotecnica Instrument) based on the Jaffe and glutamate dehydrogenase technique respectively (20), and fasting blood glucose (FBG) was also measured (21).

Urine chemistry analysis was carried out using the dipstick and urine albumin was measured using a chemistry analyzer (Le Scientific Horizon 850, USA),. Microalbumin (MALB) reagent [Biobase Industry (Shandong) Co., Ltd.], which consisted of two reagents (R1 and R2) was used in the estimation of microalbuminuria. Participants were categorized as normal, microalbuminuria and macroalbuminuria using the KDIGO criteria; <30 mg/g = normal; 30-300 mg/g = microalbuminuria; >300 mg/g = macroalbuminuria (22). The creatinine-based Chronic Kidney Disease-Epidemiology Collaboration (CKD-EPI) equation was used to estimate GFR for the participants (23, 24).

### Statistical Analysis

Data was recorded into an excel sheet and analyzed using Statistical Packaged for the Social Sciences, SPSS (SPSS version 22.0). Normally distributed continuous data were analyzed using analysis of variants (ANOVA) test and Tukey HSD *post hoc*. Data that were not normally distributed were presented as median and compared by the Kruskal Wallis test. In the case of categorical variables, the chi-square test was used to examine if there are significant associations with the indicators of interest. In addition, Spearman’s correlation was used to test for associations between appropriate variables.

Receiver Operating Characteristic Analysis (ROC) was performed to assess the diagnostic performance of appropriate variables. Statistically significant associations were determined using the conventional α = 0.05 significance level. Graphical representations of variables were presented with histograms, scatter plots, box plots, and line graphs.

## Results

### Sociodemographic Characteristics

This study involved 107 T2DM patients; 36.8% males and 63.2% females. The average age of the entire population was 58.20 ± 11.08 years with most of the participants being married (63.0%). Basic education was the highest level of education attained by most of the study participants (52.6%), followed by secondary (32.0%), and tertiary (15.5%). Christianity was the predominant religion (78.1%) among the study population. Most of the participants (52.9%) were self-employed, followed by those on pension (19.5%), the unemployed (17.2%), and public sector workers (10.3). The prevalence of microalbuminuria in this study was 79.4.4%. The results are presented in **Table 1**.

**Table 1:** Sociodemographic characteristics of the study participants.

Characteristic		Total	Non-DN	DN	P-value
		107 (100.0)	22 (20.6)	85 (79.4)	
Age		58.20 ± 11.08	61.73 ± 13.19	57.27 ± 10.35	0.093
	< 40 yrs	5 (4.7)	0 (0.0)	5 (6.0)	
	40 – 49 yrs	13 (12.3)	4 (18.2)	9 (10.7)	
	50 – 59 yrs	42 (39.6)	7 (31.8)	35 (41.7)	
	60 – 69 yrs	30 (28.3)	6 (27.3)	24 (28.6)	
	≥ 70 yrs	16 (15.1)	5 (22.7)	11 (13.1)	
Gender	Male	39 (36.8)	7 (31.8)	32 (38.1)	0.587
	Female	67 (63.2)	15 (68.2)	52 (61.9)	
Marital status	Single	23 (23.0)	5 (23.8)	18 (22.8)	0.922
	Married	63 (63.0)	14 (66.7)	49 (62.0)	
	Divorced	11 (11.0)	2 (9.5)	9 (11.4)	
	Co-habitation	1 (1.0)	0 (0.0)	1 (1.3)	
	Widowed	2 (2.0)	0 (0.0)	2 (2.5)	
Education	No education	51 (52.6)	12 (63.2)	39 (50.0)	0.351
	Basic	31 (32.0)	6 (31.6)	25 (32.1)	
	Secondary	15 (15.5)	1 (5.3)	14 (17.9)	
	Tertiary				
Religion	Christianity	82 (78.1)	17 (77.3)	65 (78.3)	0.916
	Islam	23 (21.9)	5 (22.7)	18 (21.7)	
Occupation	Unemployed	15 (17.2)	4 (20.0)	11 (16.4)	0.737
	Self-employed	46 (52.9)	10 (50.0)	36 (53.7)	
	Public sector	9 (10.3)	1 (5.0)	8 (11.9)	
	Pensioner	17 (19.5)	5 (25.0)	12 (17.9)	

DN, Diabetic Nephropathy. The test used for the association between mean age and diabetic nephropathy is independent t-test and the test used for the rest of the characteristics is chi square test.

### Anthropometric Characteristics and Medical History

The average weight, height, and BMI of the study population were 70.00kg, 161.87cm, and 26.86 kg/m^2^ respectively. Most of the respondents had a high waist-to-hip ratio (83.3%) as well as a high waist-to-height ratio (76.4%) using the WHO recommended reference range (W/H Male, 0.9 or less; female 0.85 or less, W/Ht 0.5 or less); with 90.80cm average waist circumference and 95.56cm average hip circumference. The mean FBG of the study population was 8.02 mmol/L (reference range 3.6-6.4) and the average duration of DM was 11.88 years. The majority of the participants were hypertensive (73.5%), had a family history of DM (63.8%), and also presented with high FBG levels (62.0%). The mean systolic and diastolic blood pressures were 136.53 mmHg and 87.78 mmHg correspondingly. No significant association was observed between albuminuria and medical history as well as all the anthropometric characteristics of the study participants (**Tables 2** and **3**).

**Table 2:** Anthropometric characteristics and medical history.

Characteristic	Total	Non-DN	DN 85 (79.4)		P-value
	107 (100.0)	22 (20.6)	Microalbuminuria 71 (66.4)	Macroalbuminuria 14 (13.1)	
Weight (kg)	70.00 ± 13.60	69.75 ± 10.08	69.09 ± 13.02	74.34 ± 19.66	0.487
Height (m)	1.619 ± 0.090	1.604 ± 0.116	1.617 ± 0.091	1.643 ± 0.037	0.525
BMI (kg/m^2^)	26.86 ± 5.86	27.15 ± 5.39	26.60 ± 5.88	27.45 ± 6.76	0.889
Underweight	3 (4.4)	0 (0.0)	2 (4.9)	1 (8.3)	0.419
Normal	26 (38.2)	6 (40.0)	17 (41.5)	3 (25.0)	
Overweight	18 (26.5)	3 (20.0)	9 (22.0)	6 (50.0)	
Obese	21 (30.9)	6 (40.0)	13 (31.7)	2 (16.7)	
Waist circumference (cm)	90.80 ± 25.06	78.00 ± 31.59	91.41 ± 23.92	101.73 ± 15.91	0.081
Hip circumference (cm)	95.56 ± 24.68	87.50 ± 34.28	93.88 ± 23.65	106.92 ± 13.87	0.153
Waist-to-hip ratio (WHR)	0.95 ± 0.10	0.88 ± 0.10	0.97 ± 0.10	0.94 ± 0.09	0.089
Normal	9 (16.7)	3 (30.0)	3 (9.1)	3 (27.3)	0.171
High	45 (83.3)	7 (70.0)	30 (90.9)	8 (72.7)	
Waist-to-height ratio (WHtR)	0.55 ± 0.16	0.50 ± 0.21	0.56 ± 0.16	0.60 ± 0.10	0.350
Normal	13 (23.6)	4 (36.4)	8 (24.2)	1 (9.1)	0.319
High	42 (76.4)	7 (63.6)	25 (75.8)	10 (90.9)	
FBG (mmol/L)	8.02 ± 3.39	7.67 ± 3.17	7.81 ± 3.13	9.77 ± 4.77	0.186
Normal	35 (38.0)	9 (47.4)	23 (37.1)	3 (27.3)	0.531
High	57 (62.0)	10 (52.6)	39 (62.9)	8 (72.7)	
Duration of DM	11.88 ± 7.96	11.43 ± 7.98	11.56 ± 7.26	14.00 ± 11.86	0.757
<1 year	19 (18.1)	3 (14.3)	15 (21.1)	1 (7.7)	0.753
1-4 years	29 (27.6)	7 (33.3)	19 (26.8)	3 (23.1)	
5-9 years	44 (41.9)	8 (38.1)	30 (42.3)	6 (46.2)	
≥10 years	13 (12.4)	3 (14.3)	7 (9.9)	3 (23.1)	
FH of DM					
Yes	67 (63.8)	11 (50.0)	46 (65.7)	10 (76.9)	0.235
No	38 (36.2)	11 (50.0)	24 (34.3)	3 (23.1)	
Systolic BP (mm/Hg)	136.53 ± 21.19	139.35 ± 22.80	133.74 ± 20.36	144.67 ± 21.51	0.224
Diastolic BP (mm/Hg)	87.78 ± 15.67	87.32 ± 13.56	89.92 ± 14.94	79.00 ± 19.59	0.091
Hypertension					
Yes	72 (73.5)	15 (68.2)	48 (72.7)	9 (90.0)	0.420
No	26 (26.5)	7 (31.8)	18 (27.3)	1 (10.0)	
FH of hypertension					
Yes	20 (28.2)	5 (33.3)	15 (27.8)	0 (0.0)	0.611
No	51 (71.8)	10 (66.7)	39 (72.2)	2 (100.0)	

DBP, Diastolic blood pressure; SBP, Systolic blood pressure, FBG, Fasting Blood Glucose; DM, Diabetes Mellitus; FH, Family History; BP, Blood Pressure; BMI, Body Mass Index; WC, Waist Circumference; HC, Hip circumference; WHR, Waist-to-hip-ratio; WHtR, Waist-to-height, ratio.

**Table 3:** Diagnostic performance of free light chains to detect patients with DN.

Characteristic	AUC (95% CI)	Cut-off	TP	TN	FP	FN	PPV	NPP	Sensitivity	Specificity	P-value
Albuminuria											
Kappa	0.599 (0.450-0.749)	≥16.46	56	8	7	23	88.90%	25.80%	70.90%	53.30%	0.225
Lambda	0.487 (0.321-0.653)	≥11.17	52	6	9	27	85.20%	18.20%	65.80%	40.00%	0.873
Kappa: Lambda	0.565 (0.383-0.747)	≥1.43	52	8	7	27	88.10%	22.90%	65.80%	53.30%	0.427
CKD											
Kappa	0.594 (0.478-0.710)	≥16.83	27	23	32	11	45.80%	67.60%	71.10%	41.80%	0.126
Lambda	0.601 (0.485-0.717)	≥11.17	28	23	32	10	46.70%	69.70%	73.70%	41.80%	0.098
Kappa: Lambda	0.510 (0.389-0.630)	≥1.46	24	25	30	14	44.40%	64.10%	63.20%	45.50%	0.876

TP, True Positive, FP, False Positive, NPV, Negative Predictive Value, FN, False Negative, PPV, Positive Predictive Value, TN, True Negative.

### Estimated Glomerular Filtration Rate and CKD Stages Among Study Participants

**Figure 1:**
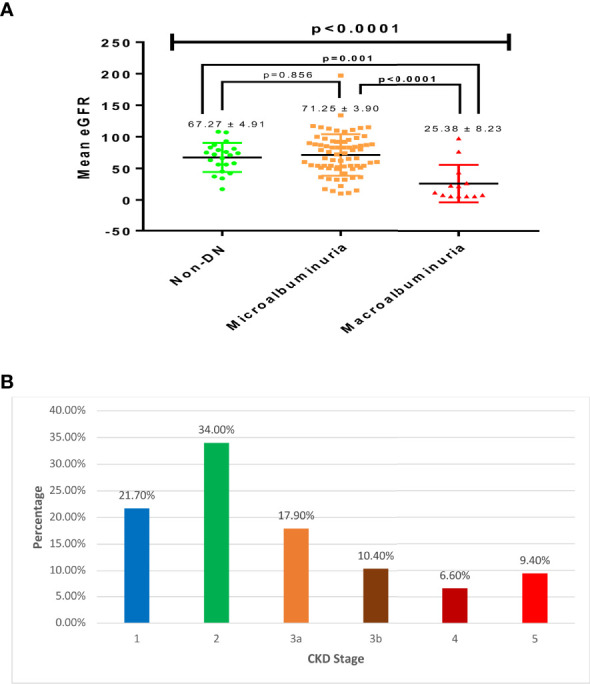
Estimated glomerular filtration rate **(A)** and CKD stages among the respondents **(B)**.

The average eGFR of the studied participants was 64.80 ± 3.29 ml/min/1.73m^2^. A significant association was observed between eGFR and DN (p<0.0001) across the studied participants (**Figure 1**). Estimated glomerular filtration rate (eGFR) was significantly lower among patients with macroalbuminuria (25.38 ± 8.23 ml/min/1.73m^2^) compared to those with microalbuminuria (71.25 ± 3.90 ml/min/1.73m^2^; p<0.0001) and non-DN (67.27 ± 4.91 ml/min/1.73m^2^; p=0.001). The prevalence of significant CKD (stages 3-5) among the study population was 44.3%; where a larger proportion of the participants (17.9%) were in stage 3a, followed by stage 3b (10.4%), stage 5 (9.4%) and stage 4 (6.6%). Most of the respondents were in stage 2 (34.0%), and stage 1 (21.7%) as shown in **Figure 1**.

### Free Light Chain Profile of Study Participants

**Figure 2:**
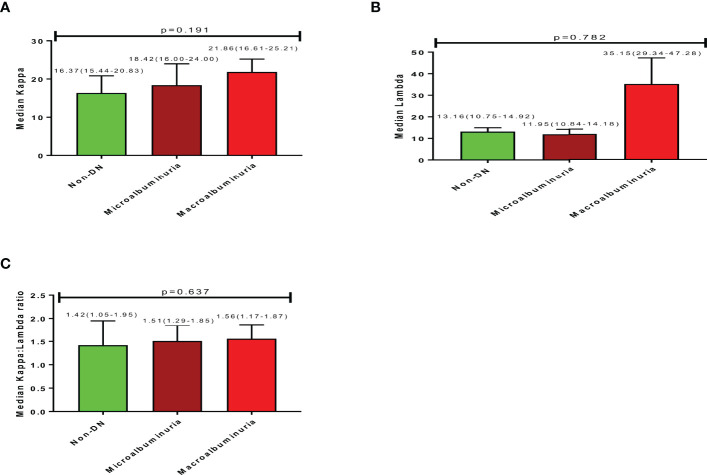
Free light chain profile of the study participants showing **(A)** (Median Kappa), **(B)** (Median Lambda), **(C)** (Median Kappa:Lambda ratio).

The median serum Kappa, Lambda, and Kappa: Lambda ratios for studied participants were 18.51(15.63-24.18), 12.19(10.84-14.48), and 1.50(1.23-1.86) respectively. An increasing trend in Kappa levels was observed with increasing nephropathy; where participants with macroalbuminuria had higher Kappa levels (21.86) followed by those with microalbuminuria (18.42) and then non-DN respondents (16.37), albeit not statistically significant (**Figure 1A**; p=0.191). A similar increasing trend was observed for K: L ratio concerning the degree of nephropathy; where participants with macroalbuminuria had a higher K: L ratio (1.56) followed by those with microalbuminuria (1.51) and non-DN respondents (1.42), with no statistical significance (**Figure 2**; p=0.637). Nevertheless, an inconsistent trend was observed for Lambda levels; where non-DN respondents had higher levels (13.16) than those with microalbuminuria (11.95), with macroalbuminuria patients recording the highest Lambda levels (35.15). No statistical significance was observed between DN and serum levels of Lambda (**Figure 2**; p=0.782).

A positive correlation was observed between albuminuria and; Kappa (rs=0.132; p=0.209), and Lambda (rs=0.076; p=0.469). However, a negative correlation was observed between albuminuria and K: L ratio (rs= -0.006; p=0.0.956). A negative correlation was observed between eGFR and; Kappa (rs= -0.187; p=0.072), Lambda (rs= -0.092; p=0.379) and K: L ratio (rs= -0.103; p=0.324).

### Diagnostic Performance of Free Light Chains

Receiver operating characteristic analysis of Kappa levels showed an optimum cut-off value of 16.46, corresponding to 70.90% sensitivity and 53.30% specificity and an area under the curve of 0.599 to predict albuminuria (i.e. microalbuminuria or macroalbuminuria). For Lambda, a cut-off value of 11.17, a sensitivity of 65.80%, a specificity of 40.00%, and an AUC of 0.487 were observed to differentiate non-DN status and those with albuminuria. Moreover, Kappa: Lambda ratio also showed a cut-off of 1.43 with a corresponding sensitivity of 65.80%, specificity of 53.30%, and an AUC of 0.565.

**Figure 3:**
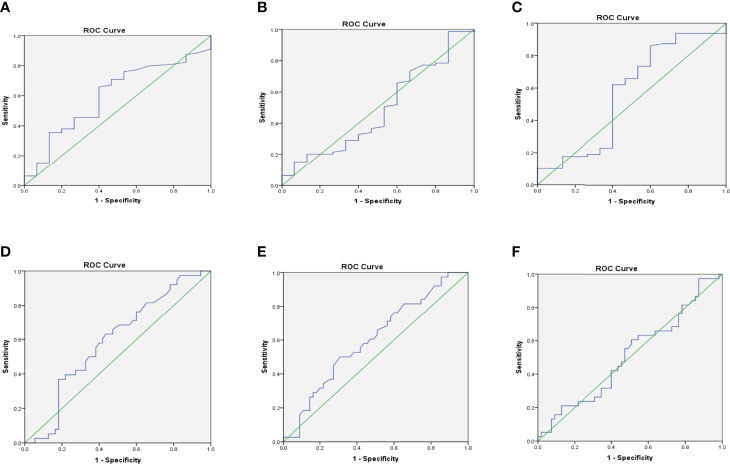
Receiver operating characteristic (ROC) curves for Kappa **(A)**, Lambda **(B)**, and K: L ratio **(C)** for predicting albuminuria; and Kappa **(D)**, Lambda **(E)**, and K: L ratio **(F)** for predicting CKD.

Kappa depicted a sensitivity of 71.10%, a specificity of 41.80%, a cut-off of 16.83, and an AUC of 0.594 to predict CKD among the studied participants. A slightly higher sensitivity of 73.70% was observed for Lambda, with corresponding specificity, cut-off, and AUC of 41.80%, 11.17, and 0.601 respectively to differentiate respondents with CKD from those without CKD. Also, the K: L ratio showed a sensitivity of 64.10%, a specificity of 63.20%, an AUC of 0.510, and a cut-off value of 1.46 to differentiate respondents with CKD from those without CKD (**Table 3**). The various ROC curves are presented in **Figure 3**.

## Discussion

This study evaluated serum free light chains as a marker of diabetic nephropathy. Our findings showed that the concentration of serum Kappa(K) and Lambda(ʎ) increased progressively with diabetic nephropathy (K: rs=0.132; p=0.209 and ʎ: rs=0.076; p=0.469), albeit not statistically significant. In addition, serum free light chains showed a positive correlation with serum creatinine and a negative correlation with estimated GFR. Our investigation of the diagnostic performance of the free light chains using ROC analysis indicated 70.90% and 53.30% sensitivity and specificity respectively for Kappa, On the other hand sensitivity of 65.80%, and specificity of 40.00% for Lambda for predicting DN.

The availability of immunoassays in the quantification of the concentration of serum free light chains has made it possible for its use in routine diagnosis and follow up (25, 26). We used these assays to measure the concentration of free light chains in a population of diabetic nephropathy patients and describe the association between serum free light chains, renal function, and disease state.

There was an increase in the median serum concentration of K with regards to the severity of nephropathy, with macroalbuminuric patients having higher levels compared with microalbuminuric and normoalbuminuric patients. On the other hand, an inconsistent trend of λ concentration was observed, in that participants without diabetic nephropathy had higher levels (13.16mg/L) compared with microalbuminuric patients, though macroalbuminuria patients recorded the highest λ levels (35.15mg/L). However, generally there was a positive correlation between serum free light chain concentration and diabetic nephropathy, although not statistically significant. Hutchison et al., in their assessment of serum free light chains in type 2 diabetic patients (South-Asian and Caucasian) observed significantly elevated concentration, even in patients with normal renal function, which is contrary to what was found in this study (19). Other studies involving CKD patients found elevated levels of free light chains across CKD stages (14, 19). Fraser et al. in a review also found that elevated concentration of serum FLC is independently associated with a higher risk of ESRD in patients with chronic kidney disease (27). The reticuloendothelial system becomes an increasingly significant route for FLC clearance when renal clearance decreases accounting for the elevated levels of free light chains associated with the severity of diabetic nephropathy seen in this current study. Excessive free light chain synthesis and subsequent filtration through renal glomeruli can damage renal tubules, resulting in tubular dysfunction, whereas renal failure from any cause can boost serum FLC concentrations due to a lower filtration rate (28).The general increase in serum FLC concentrations as seen in this study and other studies may play a pathophysiological function in the course of diabetic nephropathy (19), which will require further scientific inquiry. The findings of this study highlight the importance of further research into the role of FLC in diabetic nephropathy and its application in risk stratification.

We also observed that FLC correlated with markers of kidney function, a positive and a negative correlation was found with serum creatinine concentration and estimated GFR respectively, which was consistent with findings from other studies (12, 14, 15, 19, 29). FLC levels in the plasma rise in patients with impaired renal function (10). The current study have demonstrated that the serum concentrations of FLC in patients with diabetic nephropathy increases as renal function declines, making it a probable biomarker of diabetic nephropathy. When compared to other inflammation markers such as CRP, raised FLCs may be a better indicator of the risk of renal injury and have a greater link with renal inflammation (14, 15).

Receiver Operating Characteristic (ROC) was used to determine the diagnostic performance of FLC in detecting DN and CKD. There is a paucity of information on the use of FLC in the diagnosis of diabetic nephropathy in literature and there is little evidence of its use as a predictive technique for early detection of diabetic nephropathy. The sensitivity and specificity scores obtained with regards to FLC in this study are quite low and do not offer a good predictive value for predicting diabetic nephropathy and CKD. There is therefore the need for further studies to ascertain the role of FLC in the pathophysiology of diabetic nephropathy and its usefulness as a biomarker of early diabetic nephropathy. The study however has some limitations. Our findings, though relevant, is limited by the cross sectional design we employed instead of a case control study hence the presence of a significant number of the participants in the later stages of CKD. Again, the cross-sectional design of this study is an intrinsic limitation since it does not allow to estimate the true predictive role of these markers on the onset of DN, as patients already have diabetes mellitus.

## Conclusion

The current study observed an increasing trend in the levels of free light chains and degree of diabetic nephropathy, although not statistically significant. The exploration of serum free light chains as a better and early marker of diabetic nephropathy showed very promising results but further studies are required to elucidate its predictive value as a diagnostic tool for nephropathy.

## Data Availability Statement

The raw data supporting the conclusions of this article will be made available by the authors, without undue reservation.

## Ethics Statement

The studies involving human participants were reviewed and approved by the Institutional Review Board of the Research and Development Unit of Komfo Anokye Teaching Hospital (KATH) Kumasi, Ghana, with reference KATHIRB/AP/080/20. The patients/participants provided their written informed consent to participate in this study.

## Author Contributions

ES, WO, PO and RE designed the study and supervised the work. ES, FS, PO and SD were in charge of major parts of technical aspects of work and participated in the writing of the manuscript. ES, RB and PO participated in the technical work and participated in the interpretation of data. ES, RE, FS participated in the manuscript writing. All authors read and approved the final manuscript. All authors contributed to the article and approved the submitted version.

## Conflict of Interest

The authors declare that the research was conducted in the absence of any commercial or financial relationships that could be construed as a potential conflict of interest.

## Publisher’s Note

All claims expressed in this article are solely those of the authors and do not necessarily represent those of their affiliated organizations, or those of the publisher, the editors and the reviewers. Any product that may be evaluated in this article, or claim that may be made by its manufacturer, is not guaranteed or endorsed by the publisher.
